# Schema modes and their associations with emotion regulation, mindfulness, and self-compassion among patients with personality disorders

**DOI:** 10.1186/s40479-021-00160-y

**Published:** 2021-06-10

**Authors:** Ella Salgó, Bettina Bajzát, Zsolt Unoka

**Affiliations:** Department of Psychiatry and Psychotherapy, Semmelweis University, Budapest, Hungary

**Keywords:** Emotion regulation, schema therapy, mindfulness, self-compassion, Externalizing, p factor, Cognitive Emotion Regulation Questionnaire, Difficulties in Emotion Regulation Scale, Self-Compassion Scale, Five Facet Mindfulness Questionnaire, canonical correlation analyses

## Abstract

**Background:**

The current study's goal was to examine the multivariate patterns of associations between schema modes and emotion regulation mechanisms in personality disorders. Schema modes are either integrated or dissociative states of mind, including intense emotional states, efforts to regulate emotions, or self-reflective evaluative thought processes. Exploring the multivariate patterns of a shared relationship between schema modes and emotion regulation strategies may lead to a better understanding of their associations and a deeper understanding of the latent personality profiles that organize their associations in a mixed personality disorder sample.

**Methods:**

Patients who have personality disorders (*N* = 263) filled out five different self-report questionnaires, out of which four measured adaptive and maladaptive emotion-regulation strategies (Cognitive Emotion Regulation Questionnaire, Difficulty of Emotion Regulation Scale, Five Factor Mindfulness Questionnaire, Self-Compassion Scale), and the fifth one assessed schema modes (Schema Mode Inventory). We conducted canonical correlation analysis in order to measure the multivariate patterns of associations between the 26 emotion regulation and the 14 schema mode subscales.

**Results:**

We found strong multivariate associations between schema modes and emotion regulation strategies. Collectively, the full model based on all canonical variate pairs was statistically significant using the Wilks’s Λ = .01 criterion, F (364,2804.4) = 3.5, *p* < .001. The first two canonical variate pairs yielded interpretable squared canonical correlation (Rc^2^) effect sizes of 74.7% and 55.8%, respectively. The first canonical variate pair represents a general personality pathology variable with a stronger weight on internalization than externalization, and bipolarity in terms of adaptive vs. non-adaptive characteristics. We labeled this variate pair "Adaptive/Non-Adaptive." The second canonical variate pair, labeled "Externalizing", represents externalizing schema modes and emotion regulation strategies.

**Conclusion:**

Using a multivariate approach (CCA), we identified two independent patterns of multivariate associations between maladaptive schema modes and emotion regulation strategies. The Adaptive/Non-Adaptive general personality pathology profile and the Externalizing personality pathology profile may lead to a deeper understanding of personality disorders and help psychotherapists in their conceptualization in order to design the most appropriate interventions.

**Supplementary Information:**

The online version contains supplementary material available at 10.1186/s40479-021-00160-y.

## Introduction

### Theoretical Background

Schema Therapy (ST) was developed by J.E. Young [1] to treat personality disorders and chronic Axis I disorders. A central element of ST is the concept of schema mode, which is a recurring pattern of intense emotions, thoughts, feelings, and behavior that is active at a given point in time [2]. Modes are triggered by the activation of early maladaptive schemas, which are dysfunctional emotional and cognitive patterns that were established in childhood and are reemerging throughout life. Adverse early experiences (e.g.: childhood abuse, emotional neglect, lack of secure attachment) and the frustration of basic childhood needs (safety and attachment, autonomy, freedom to express feelings, spontaneity, boundaries) lead to the development of maladaptive schemas about one’s self, one’s relationships to others and the world. When a maladaptive schema gets activated, the associated difficult emotions arise with it. In order to deal with the painful emotions, different coping strategies are developed (avoidance, surrender, overcompensation). Schema modes are the combinations of the activated schemas and coping strategies, being the momentary reflections of the individual’s emotional, cognitive and behavioral state. The main difference between schemas and schema modes is that modes may shift from one into another very quickly and abruptly, explaining the sudden emotional and behavioral changes observable in patients with severe PD [3]. When maladaptive schema modes are active, the person is in a dissociative state of mind. Schema modes are organized into four categories: child modes, coping modes, parent modes, and the Healthy Adult mode. Modes can be adaptive (the Healthy Adult and the Happy Child mode) or maladaptive (every other mode). Innate child modes, activated by unmet basic emotional needs, are characterized by feelings of sadness, shame, anger, and vulnerability. Coping modes are maladaptive regulatory strategies that mitigate the effect of the emotional response to unmet needs in the short run, but cause dysfunctional emotion regulation in the long term, since they do not lead to the satisfaction of core needs. For example, by avoiding certain situations, or by overcompensating the triggered maladaptive schemas. Parent modes are characterized by internalized self-destructive messages that generate contempt toward core emotional needs. The adaptive Healthy Adult mode integrates adaptive thoughts, behaviors, and cognitions leading to well-functioning emotion regulation, while the Happy Child is a mode where basic emotional needs are met, and the person feels loved, accepted and agented. Young's original concept consisted of 10 schema modes. Since the original schema mode concept had been developed, altogether, 22 modes have been defined and hypothesized to be prominent in specific PDs [4]. However, in this research, we use the 14-factor model of the Schema Mode Inventory [5]. (See Table 1.)

**Table 1:** Schema modes

Categories	Schema Modes
Healthy mode	Healthy Adult
Child modes	Happy Child
	Vulnerable Child
	Impulsive Child
	Angry Child
	Undisciplined Child
	Enraged Child
Dysfunctional coping modes	Compliant Surrenderer
	Detached Protector
	Detached Self-Soother
	Self-Aggrandizer
	Bully and Attack
Parent modes	Punitive Parent
	Demanding Parent

### Emotion regulation in Schema Therapy

In Schema Therapy, emotion dysregulation is conceived due to adverse early experience, and schema modes are intense dysregulated emotional states, internalized critical messages, and maladaptive copings related to basic unmet needs. Higher levels of Early Maladaptive Schemas are connected to more severe emotional dysregulation [1]. Negative emotion regulation mediates the relationship between Early Maladaptive Schemas and psychopathological symptoms [6]. According to the concept of Dadomo et al. [7], every schema mode can be associated with specific dysregulated emotions and dysregulatory strategies. Child modes are associated with specific emotions such as anger, shame, and sadness. Parent modes are characterized by, for example, self-reflective emotions, such as excessive guilt, shame, and contempt, while healthy modes are defined by happiness and the feeling of being loved. Dysregulatory strategies that are associated with the different modes are, for example, self-blame, blaming others in the Vulnerable and Angry Child Modes, isolation, and devaluing others in the Detached Protector and Overcompensator modes, or self-punishment in the Maladaptive Parent Modes.

The concept of emotion regulation refers to a series of conscious and non-conscious strategies that are aimed to modulate the onset, intensity, duration, and quality of emotions [8]. Emotion regulation does not only mean diminishing negative emotions. It also entails the acceptance and awareness of negative and positive emotions in the given context [9]. According to Gross's process model, emotions can become dysregulated, when a patient fails to use an appropriate regulatory strategy. The model discusses five different regulatory processes that can be effective in varying stages of emotional experience: situation selection, situation modification, attention deployment, cognitive change, and response modification. In the last 20 years, based on Gross’s model, several studies showed in healthy populations that cognitive reappraisal correlates positively with well-being and negatively with symptoms of psychopathology [8, 10, 11], while suppression (a response-focused strategy) is positively associated with depressive symptoms and negatively with satisfaction in interpersonal relations [12, 13]. An opposing theory, the experiential-dynamic emotion-regulation model (EDER) developed by Grecucci et al. [14], proposes that emotions are not inherently dysregulated in lack of regulatory strategies. This model supposes that dysregulation derives from the combination of emotions plus conditioned anxiety or secondary defensive coping mechanisms. The EDER model is grounded in affective neuroscience and modern psychodynamic psychotherapy, and opposes the assumption of cognitive regulation models that cognitive appraisals occur before emotional reactions, stating that emotion has a neurobiological primacy over cognition in terms of temporal dynamics and anatomical circuitry as well. While based on Gross's model, the clinician needs to promote better regulatory strategies, the EDER model demands clinicians to regulate the dysregulating anxiety or to restructure coping mechanisms to help the patient express the underlying emotions. There are several other approaches of emotion regulation (e.g. appraisal theory, constructionism), but they all agree that a wide range of psychopathological symptoms can be described as the failure of emotion regulation. In agreement with the EDER model's approach, schema mode therapy aims to increase the presence of the Healthy Adult mode by removing or mitigating the effect of dysregulatory coping modes and critical parent modes so that the healthy affective response can be restored. The concepts of emotion dysregulation and schema modes are thus overlapping, since maladaptive schema modes are intense dysregulated emotional states, self-reflective dialogues or reactive coping behaviors, and both phenomena are aimed to modulate the subjective experience. It is important to note that according to schema theory, an unmet need induces negative emotions, and all emotion regulation that is adaptive in the long term aims to satisfy unmet needs.

### Cognitive Emotion Regulation

Cognitive emotion regulation means the conscious, cognitive process of coping with emotionally triggering information [15]. Cognitive emotion regulation is different from cognitive coping, because cognitive emotion regulation theory considers thinking and acting two different processes, therefore separates cognitive strategies from behavioral ones [16]. The Cognitive Emotion Regulation Questionnaire (CERQ) refers exclusively to an individual's cognitions after having experienced a threatening or stressful life event [17]. There are altogether nine adaptive and maladaptive cognitive regulation strategies included in the questionnaire (Self-blame, Other-blame, Rumination, Catastrophizing, Putting into Perspective, Positive Refocusing, Positive Reappraisal, Acceptance, and Refocus on Planning). According to a meta-analysis [18], maladaptive strategies are more strongly connected to symptoms of mental disorders than adaptive strategies, and mood-related disorders are more strongly connected to emotion regulation strategies than other disorders. In a study by van Wijk-Herbrink et al. [19], the principal component analysis of the CERQ found three higher-order domains in a sample of patients with personality disorders; adaptive coping, maladaptive coping, and external attribution. We do not know about any previous study that investigated the associations between cognitive emotion regulation strategies measured by CERQ and schema modes. Based on van Wijk-Herbrink et al.’s study, maladaptive emotion regulation scales of CERQ will be associated with maladaptive schema modes, adaptive emotion regulation scales of CERQ will be associated with adaptive schema modes, and external attribution will be associated with externalizing schema modes: Angry/Rageful Child modes, Punitive Parent and Bully Attack modes.

### The Difficulties in Emotion Regulation Scale's (DERS) concept of emotion regulation

The development of DERS [9] was based on the concept that emotion regulation entails four main facets: (a) awareness and understanding of emotions, (b) acceptance of emotions, (c) ability to control impulsive behaviors and behave in accordance with desired goals when experiencing negative emotions, and (d) ability to use situationally appropriate emotion regulation strategies flexibly to modulate emotional responses as desired in order to meet individual goals and situational demands. This model is far broader than the classic emotion regulation concept of Gross, and was designed to assess trait-level perceived emotion regulation ability in a clinical-contextual framework. Higher scores on the measure indicate greater dysfunctionality or dysregulation. According to a review, maladaptive emotion regulation strategy use and overall emotion dysregulation in different psychopathologies, measured by DERS among other scales, were found to significantly decrease following psychotherapeutic treatment [20]. Our hypothesis is that DERS scales will have positive associations with maladaptive schema modes and will be negatively associated with adaptive schema modes.

### Mindfulness as a form of emotion regulation

Mindfulness is a non-judgmental, present-focused state of mind in which thoughts, perceptions, and feelings are accepted and purposefully brought into attention [21]. The lack of acceptance as an emotion regulation strategy was demonstrated in several disorders, e.g. in generalized anxiety disorder [22], panic disorder [23], heroin-addiction [24] and in borderline personality disorder [25]. Suppression and avoidance are maladaptive reactions and risk factors in the development of distress experienced in depression and anxiety disorders, and might lead to maladaptive behavior e.g. drug abuse [26, 27]. The relationship between personality organization level and borderline-depressive symptoms is mediated by rumination [28]. Research proved that early maladaptive schemas are negatively associated with mindfulness and self-compassion [29]. Another study [30] demonstrated that there are strong negative associations between trait mindfulness and early maladaptive schemas among adult men seeking residential substance abuse treatment. Lower levels of mindfulness and self-compassion mediate the relationship between early maladaptive schemas and psychological distress. The effect of early maladaptive schemas on behavior is mediated by schema modes [31]. Based on this finding it can be hypothesized that the activation of schema modes is related to the level of mindfulness and self-compassion. Specifically, Healthy Adult mode will have a positive correlation and maladaptive schema modes will have negative correlations with mindfulness.

### Self-compassion's role in emotion regulation

Self-compassion, according to Neff's concept [32], involves three elements: (a) treating oneself with gentleness and acceptance rather than criticism and belittling (i.e., Self-Kindness vs. Self-Judgment), (b) acknowledging failures or imperfections as common human experiences rather than unique and isolated to the individual (i.e., Common Humanity vs. Isolation), and (c) finding balance between non-judgmental appraisal and the suppression of emotions rather than pessimistic self-victimization (i.e., Mindfulness vs. Overidentification). Self-compassion facilitates the adaptive handling of emotions instead of having negative, punitive thoughts. Moreover, self-compassion creates an emotional distance from stressful events and enables us to see the context and the negative event more realistically, which is a common factor of self-compassion and mindfulness [33]. Punitive and Demanding Parenting Modes are characterized by overidentification, isolating self-judgment. Our hypothesis is that Self-Kindness, Common Humanity and Mindfulness will have positive associations with the Healthy Adult Mode, while Self-Judgment, Isolation and Over-identification will have positive associations with the Punitive and Demanding Parenting Modes, and vice versa.

### Goals and Hypothesis

The aim of the present research was to examine the multivariate relationships between the set of schema modes and the set of emotion regulation strategies in personality disorders. Since neither schema modes nor emotion regulation strategies are independent of each other, we applied a multivariate method, Canonical Correlation Analysis (CCA), to evaluate the simultaneous relationship between schema modes measured by the SMI and emotion regulation strategies measured by the scales of CERQ, DERS, FFMQ, SCS. In CCA, the relationship between two sets of variables is studied by creating derived variables (i.e., latent variables or in the terminology of CCA “variates”) separately in the two variable sets that are linear composites of the original variables. The objective of this procedure is to obtain as high a correlation as possible between the derived variables from the first and second variable set (i.e., between the pairs of variates formed from the two sets, respectively). This technique, in mathematical sense represents an optimal linear method to investigate interset association, since components from the two variable sets are extracted jointly in a way maximizing the correlation between the components [34].

Using this procedure, the observed schema mode variables as well as the emotion regulation variables are simultaneously decomposed into ’factors’ (canonical variate pairs) which maximally correlate with each other, but are perfectly uncorrelated with the subsequent canonical variates yielded by the analysis (i.e., the 1st canonical factor pair, including canonical variate 1 from the schema mode set and canonical variate 1 from the emotion regulation set will correlate with each other, but have no correlation with the subsequent canonical factor pairs).

The overall aim of our study was to investigate the question of whether schema modes were related to emotion regulation strategies.

1. More specifically, based on the aforementioned literature our assumption was that the first canonical variate pair would include most of the associations between schema modes and emotion regulations in one general personality pathology model. We also expected that the consecutive canonical variate pairs would represent specific associations either along the adaptive-maladaptive axes or along internalizing-externalizing axes or would have a more specific variate pair that represent compulsivity, as it was found in a hierarchical factor analysis of schema modes [35].
2. We also hypothesized that adaptive schema modes would be positively associated with other adaptive schema modes and adaptive emotion regulation strategies while negatively associated with maladaptive schema modes and non-adaptive emotion regulation strategies. Furthermore, maladaptive schema modes would have positive associations with non-adaptive emotion regulation strategies.
3. Based on van Wijk-Herbrink et al.'s study, we assumed that CERQ scales would form three distinct groups: maladaptive (catastrophizing, rumination, and blaming others), adaptive (positive reappraisal, putting into perspective, positive refocusing, acceptance, and refocus on planning), and external attribution (negative loading for self-blame and positive loadings for positive refocusing and blaming others) emotion regulation.

## Method

### Subjects and Procedure

Subjects were participants of an at least 4 week long psychotherapy program at Semmelweis University's Department of Psychiatry and Psychotherapy between 2017 and 2019. Data were analyzed from 263 subjects diagnosed with different personality disorders. 180 were female (68.4%) and 83 were male (31.6%), with a mean age of 36.6 years (SD=12.6, range = 18-72). With respect to educational level, 0.8 % completed the first 6 years of primary school, 19.4 % did not complete a secondary education, 27.8 % completed secondary school, 6.5 % dropped out of college, 8.7 % completed vocational studies, 14.1 % were college or university students, 4.6 % dropped out of university, while 16.7 % obtained university degrees, in case of 1,5 % of participants data about education was missing. The distribution of clinical diagnosis was the following: 30,99 % borderline, 25,82 % depressive, 24,4 % avoidant, 17,84 % obsessive-compulsive, 17,31% dependent, 15,96 % paranoid, 12,67 % histrionic, 13,14 % passive-aggressive, 9,86 % narcissistic and 2,8 % schizotypal personality disorder, in case of 19% of participants data about SCID II diagnostic interviews was missing. All subjects received information about the research and signed the informed consent sheet. Their anonymity was secured. Participants were diagnosed with SCID-II interviews and filled out questionnaires online. The research procedure was approved by the Semmelweis University Regional and Institutional Committee of Science and Research Ethics.

### Self-Reported Questionnaires

#### Young Schema Mode Inventory (YSI)

The 124-item Young Schema Mode Inventory [5] was developed to assess the presence of 14 schema modes. The model consists of five child modes, five dysfunctional coping modes, two dysfunctional parent modes, and the adaptive Healthy Adult mode. Cronbach's α coefficients of the schema mode subscales in this study ranged from (.62) to (.92). Answers are rated on a 6-point Likert scale. The Hungarian adaptation of YSI was applied in our study [36].

#### Materials measuring emotion regulation strategies

1. The Cognitive Emotion Regulation Questionnaire (CERQ) is a 36-item questionnaire evaluating cognitive emotion regulation strategies used after having experienced negative life events or situations [37]. It measures nine different cognitive coping strategies: Self-Blame, Other-Blame, Rumination or focus on thought, Catastrophizing, Putting into Perspective, Positive Refocusing, Positive Reappraisal, Acceptance and Refocus on Planning. Cronbach's α coefficients of the subscales in this study ranged from (.59) to (.86). A 5-point Likert scale is used to measure cognitive emotion regulation strategies, ranging from 1 (almost never) to 5 (almost always). The questionnaire was used in its Hungarian version [38].

2. To assess the degree of difficulty of emotion regulation, the Difficulties in Emotion Regulation Scale (DERS) [9] was implemented in its Hungarian form [39]. The 36 items of DERS are organized into a 6-factor structure: Non-acceptance of emotional responses, Difficulty engaging in goal-directed behavior, Impulse control difficulties, Lack of emotional awareness, Limited access to emotion regulation strategies and Lack of emotional clarity. Cronbach's α coefficients of the subscales in this study ranged from (.55) to (.88). DERS uses a 5-point Likert scale.

3. The Self-Compassion Scale (SCS), developed by Dr. Kristine Neff [40], is used to measure self-compassion, which is by definition compassion turned inward, and refers to how we relate to ourselves in instances of perceived failure, inadequacy or personal suffering [41]. The scale consists of 26 items rated on a 5-point Likert scale. Its three subscales are Self-Kindness versus Self-Judgment, a sense of Common Humanity versus Isolation, and Mindfulness versus Over-identification. Cronbach's α coefficients of the subscales in this study ranged from (.50) to (.86). The Hungarian version of SCS was used in our research [42].

4. The Five Facet Mindfulness Questionnaire is a 39-item inventory that investigates the five main aspects of mindfulness on a 5-point Likert scale: Observation, Description, Mindful Actions, Non-Judgmental Inner Experience and Non-Reactivity [43]. Cronbach's α coefficients of the subscales in this study ranged from (.71) to (.91). The Hungarian adaptation of the scale was used in this study [44].

### Statistical Analysis

In order to examine the multivariate relationships between schema modes and emotion regulation skills we applied canonical correlation analysis (CCA). CCA is a multivariate analysis of „interset association”, allowing to uncover latent canonical variate pairs that represent the maximized linear relationship between two sets of variables [45]. A CCA using nine CERQ, six DERS, five FFMQ, and six SCS sub-scales as independent variables of each of the 14 SMI schema modes as dependent variables was conducted. Our design is cross-sectional, therefore we do not assume a causal relationship between the two sets of variables (and consequently the designation of the variables as independent or dependent is interpretable only in statistical sense) [45]. CCA can examine the sets of emotion regulation variables and the set of schema mode variables together, not only in terms of whether the variables in the two sets are correlated but also in terms of shared correlation within each variable set. In view of our hypothesis, in which more than one emotion regulation variable may associate with more than one schema mode, CCA offers a powerful approach for analyzing our data. We used Sherry and Henson’s [46] CCA syntax written for SPSS (2005), and our interpretation of our results follows their pieces of advice.

## Results

### Means, standard deviations and intercorrelations

Means, standard deviations, and intercorrelations for the variables of interest are provided in Additional file 1 (See Additional file 1).

### Canonical Correlation Analysis

A CCA was conducted using nine CERQ, six DERS, five FFMQ, six SCS sub-scales as independent variables, and the 14 SMI schema modes as dependent variables to analyze the multivariate shared relationship between schema modes and emotion regulation strategies. Our analysis yielded fourteen canonical variate pairs. There are as many canonical variate pairs in a CCA as variables in the smaller of the two variable sets, namely the 14 schema modes. The first two canonical variate pairs yielded interpretable squared canonical correlation (Rc^2^) effect sizes of 74.7% (Eigenvalue = 2,96) and 55.8% (Eigenvalue = 1,26), respectively. Squared canonical correlation (Rc^2^) represents the proportion of variance shared by the variate pair. Because a variate pair represents the observed schema mode and emotion regulation variables, the Rc^2^ indicates the amount of shared variance between the variable sets. The rest of the subsequent variate pairs (n=12 pairs) were omitted from interpretation as they explained less than 6% of the maximum shared variance between the two variables sets included in the analysis.

Collectively, the full model across all variates was statistically significant using the Wilks’s Λ = .01 criterion, F (364,2804.4) = 3.5, *p* < .001. Because Wilks's Λ represents the variance unexplained by the model, 1 – Λ yields the full model effect size in an *r*^2^ metric (Henson, 2006).

Table 2 presents structure coefficients for Canonical Variate Pairs 1 and 2. The squared structure coefficients are also given, as well as the communalities (h2) across the two variate pairs for each variable. A structure coefficient (rs) is the bivariate correlation between an observed variable and a variate, and in our case, between a schema mode scale variable and the canonical variate score for the variable's set, the schema mode variate. Our interpretation of the variate pairs is based on the structure coefficients. Squared canonical structure coefficients indicate the proportion of variance an observed variable linearly shares with the variates generated from the observed variable's set.

**Table 2:** Canonical variate pairs of schema mode and emotion regulation variables

	1 Adaptive/Nonadaptive variate pair		2 Externalizing variate pair		
Variable	***rs***	***rs***^***2***^ ***(%)***	***rs***	***rs***^***2***^ ***(%)***	h^**2**^ (%)
	**1**^**ST**^ **Canonical variate pair: Schema mode variate**		**2**^**nd**^ **Canonical variate pair: Schema mode variate**		
Vulnerable Child	**0.80**	65.6	0.12	1.6	67.1
Impulsive Child	**0.55**	30.7	**0.68**	45.9	76.6
Angry Child	**0.51**	25.9	**0.64**	41.5	67.4
Undisciplined Child	**0.54**	29.6	**0.41**	16.5	46.1
Enraged Child	**0.39**	14.9	**0.72**	51.3	66.2
Happy Child	**-0.58**	33.6	0.05	0.3	33.9
Compliant Surrender	**0.69**	47.4	-0.1	1.1	48.5
Detached Protector	**0.75**	57.2	0.19	3.6	60.8
Detached Self-Soother	0.19	3.4	-0.04	0.1	3.5
Self-Aggrandizer	**0.31**	9.4	**0.46**	21.5	30.9
Bully Attack	**0.33**	10.6	**0.51**	26.5	37.2
Punitive Parent	**0.78**	47.8	-0.15	2.3	50.1
Demanding Parent	**0.49**	24.4	-0.27	7.6	31.9
Healthy Adult	**-0.71**	50.6	-0.1	1.0	51.6
Adequacy (schema modes)		32.2		15.8	
*R*c^2^		74.7		55.8	
Adequacy (emotion regulation)		24.1		5.6	
	**1**^**ST**^ **Canonical variate pair: Emotion regulation variate**		**2**^**nd**^ **Canonical variate pair: Emotion regulation variate**		
CERQ Self-blame	**0.49**	24.0	**-0.34**	15.52	27.4
CERQ Acceptance	0.08	0.6	0.09	0.89	0.7
CERQ Rumination	**0.31**	9.9	0.18	3.26	13.2
CERQ Positive refocusing	**-0.47**	22.1	-0.07	0.00	12.6
CERQ Planning	**-0.40**	16.0	-0.05	0.23	16.2
CERQ Positive reappraisal	**-0.43**	18.2	0.04	0.19	18.4
CERQ Perspective taking	**-0.30**	9.1	-0.06	0.40	9.5
CERQ Catastrophizing	**0.40**	15.7	0.16	2.45	27.2
CERQ Other-blame	0.13	0.2	**0.59**	35.05	35.3
DERS Nonaccept	**0.53**	27.7	-0.20	4.09	31.8
DERS Goals	**0.60**	36.4	**0.29**	8.45	44.8
DERS Impulse	**0.60**	35.5	**0.54**	28.93	64.5
DERS Awareness	**0.34**	11.7	-0.06	0.41	12.2
DERS Clarity	**0.57**	32.1	0.05	0.23	32.3
DERS Strategy	**0.72**	51.2	0.08	0.68	51.2
FFMQ Observe	0.05	0.0	0.04	0.18	0.5
FFMQ Describe	**-0.61**	37.5	0.11	1.29	38.8
FFMQ Mindful Action	**-0.76**	58.8	-0.31	9,68	68.5
FFMQ Nonjudge	**-0.65**	42.4	0.11	1.23	43.7
FFMQ Nonreact	-0.21	4.3	-0.14	1.97	6.2
SCS Self-Kindness	**-0.63**	39.6	**0.29**	8.4	48
SCS Self-Judgment	**0.71**	49.7	**-0.30**	9.09	58.8
SCS Common Humanity	**-0.38**	14.8	0.05	0.28	15
SCS Isolation	**0.60**	36.2	-0.04	0.14	36.4
SCS Mindfulness	**-0.47**	22	-0.07	0.49	22.5
SCS Over-Identification	**0.57**	32.7	**0.35**	12.43	45.1

Coefficient = standardized canonical variate coefficient; rs = structure coefficient; rs^2^ = structure coefficient squared or variance explained; h^2^ = communality coefficient. An adequacy coefficient indicates how adequately the synthetic scores on a variate do at reproducing the variance in a set of variables. It is the mean of the squared structure coefficients on the variable. Noteworthy structure coefficients, with an rs-value of >0.25, are in bold type.

The first Canonical Variate Pair, the' Adaptive-Nonadaptive' canonical variate pair, is “bipolar”, i.e., it includes positive and negative structure coefficients. For the First Variate coefficients, the relevant schema mode variables with the positive signs are primarily Vulnerable Child, Compliant Surrender, Detached Protector, Punitive Parent, and secondarily Impulsive Child, Angry Child, Undisciplined Child, Enraged Child, Self-Aggrandizer, Bully Attack, Demanding Parent. Happy Child and Healthy Adult are inversely related to the other schema modes. The squared structure coefficients support this conclusion. The emotion regulation variable set in the first canonical variate pair is also bipolar with positive and negative signs of structure coefficients. Variates with the positive sign are DERS Strategy, SCS Self-Judgment, SCS Isolation, DERS Goals, DERS Impulse, DERS Clarity, SCS Over-Identification, DERS Nonaccept, CERQ Self-blame, CERQ Catastrophizing, DERS Awareness, CERQ Rumination in the order of the magnitude of structure coefficients. Variates with the negative sign are FFMQ Mindful Action, FFMQ Nonjudge, SCS Self-Kindness, FFMQ Describe, SCS Mindfulness, CERQ Positive refocusing, CERQ Positive reappraisal, CERQ Planning, SCS Common Humanity, CERQ Perspective taking in the order of the magnitude of structure coefficients. Adaptive schema modes, Healthy Adult and Happy Child, and adaptive emotion regulation skills have negative signs in the two sides of the variate pair, and maladaptive schema modes and non-adaptive emotion regulation skills have a positive sign in both sides of the first variate pair.

The Second Canonical Variate Pair, labeled „Externalisation”, is orthogonal to the first canonical variate pair. For the second canonical variate pair, the relevant schema mode variates with the positive signs are Enraged Child, Impulsive Child, Angry Child, Undisciplined Child, Self-Aggrandizer, and Bully Attack. The emotion regulation variate set in the second canonical variate pair is bipolar with positive and negative signs of structure coefficients. Variates with the positive signs are CERQ Other-blame, DERS Impulse, SCS Over-Identification, DERS Goals, SCS Self-Kindness in the order of the magnitude of structure coefficients. Variates with the negative signs are CERQ Self-blame and SCS Self-Judgment in the order of the magnitude of structure coefficients. Anger, rage, undisciplined and impulsivity-related child modes, and overcompensation schema modes with positive signs are on the second variate pair's schema mode side. High blaming-others with low self-blaming and high self-kindness with low self-judgment with high over-identification, impulsivity, goals are on the second variate pair's emotion regulation side.

In order to measure the internal consistency of the subscales of all the questionnaires, Cronbach's alpha tests were calculated. Cronbach's alpha scores range between 0.50 (SCS Self-Judgment subscale) – 0.92 (YSI Vulnerable Child subscale). Out of the 40 subscales 34 are above the 0.7 Cronbach's alpha score, which is the acceptable level of internal consistency. The scales that are under the 0.7 score are the following: Acceptance (.59) in CERQ, Difficulties in goal-directed behavior (.56) in DERS, Self-Judgment (.50), Over-Identification (.66) and Mindfulness (.66) in SCS, and Compliant Surrenderer (.69) in YSI.

## Discussion

Our study examined the multivariate patterns of the relationships between schema modes and emotion regulation strategies in personality disorders. Our results supported our general hypothesis that schema modes and emotion regulation strategies are associated.

Our first more specific hypothesis was that the first canonical variate pair would include most of the associations in one general personality pathology model; this assumption was confirmed. The consecutive canonical variate pairs would represent specific associations either along the adaptive-nonadaptive axes or along internalizing-externalizing axes, or would have a more specific variate pair representing compulsivity, which was partially confirmed. The first canonical variate pair contains the adaptive-nonadaptive axes and a mixed internalization-externalization general personality pathology dimension. Furthermore, our second canonical variate represents the externalizing personality pathology dimension. Finally, an independent compulsivity variate did not appear in our results.

Our second specific hypothesis, that adaptive schema modes would be positively associated with other adaptive schema modes and adaptive emotion regulation strategies, while negatively associated with maladaptive schema modes and non-adaptive emotion regulation strategies, was also confirmed. Furthermore, the hypothesis that maladaptive schema modes would have positive associations with non-adaptive emotion regulation strategies was also supported by our results.

Two interpretable canonical variate pairs emerged, and they give a deeper understanding of latent variables that organize the schema mode and emotion regulation scales in two orthogonal canonical variate pairs in a mixed personality disorder sample. These two orthogonal variate pairs represent two main higher-order structures of psychopathology. The first canonical variate pair represents a general personality pathology latent variable with a stronger accent on internalization than externalization and bipolarity regarding adaptive and non-adaptive characteristics. We labeled this variate pair "Adaptive/Nonadaptive." The second canonical variate pair that we labeled "Externalizing" represents a particular personality profile. As a primary latent variable, the general personality pathology captures the common variance of the transdiagnostic schema mode [47], and emotion regulation variables shared across personality disorder diagnoses [20, 48] General personality pathology, the 'g or p factor' is a recurrent finding in different analyses of a broad spectrum of psychopathology in a mixed sample of mental disorders [49–51] and samples of personality disorders [52–54]. Some authors hypothesize that neuroticism, a tendency to experience negative emotions, which has the strongest association with internalization and externalization pathology, overlaps with the 'p factor' [51]. Two papers also reported the 'p factor' in investigating the hierarchical structure of schema concepts. Bach et al. [55] found a one-component model of general maladaptivity in analyzing early maladaptive schemas' hierarchical structure. Jacobs et al. [35] found an unidimensional solution for schema modes' factor analyses, labeled Personality Pathology, and all maladaptive schema modes loaded onto it positively while the two adaptive schema modes loaded negatively. This finding is similar to the schema mode side of the Adaptive-Non-Adaptive canonical variate pair in our study. At the second level of the hierarchical structure of schema modes, Jacobs et al. [35] found two factors: internalization and externalization. The latter externalization factor was defined by the Impulsive Child, Enraged Child, Self-Aggrandizer, and Bully Attack modes, similarly to the 2. schema mode variate (Externalizing) in our results.

According to our second specific hypothesis, the Healthy Adult and Happy Child modes and the adaptive emotion regulations have the same sign, indicating that they are all positively related, while the maladaptive schema modes and non-adaptive emotion regulations are inversely related to them. Schema therapy's main target is helping patients recognize, validate, express their own core emotional needs, and find adaptive ways to have their emotional needs met. According to this conceptualization, in Healthy Adults mode the person can realize the aforementioned tasks by dealing with emotions, solving problems, and creating healthy relationships. Meanwhile, he is aware of his needs, possibilities, and limitations and acts by following their values, needs, and goals. Actions generated in the Healthy Adult mode lead to the more frequent experience of the Happy Child mode and the experience of core emotional needs being met, leading to joy, fun, play, and spontaneity [19]. The emotion regulation strategies positively related to the Healthy Adult and Happy Child modes in the first canonical variate pair may lead to a deeper understanding of the patterns of associations between adaptive schema modes and emotion regulation. Based on our results, we can say that adaptive schema modes were strongly associated with acting with awareness (FFMQ Mindful Action); not judging of inner experience, that is taking a non-evaluative stance toward thoughts and feelings (FFMQ Nonjudge); being able to be caring and understanding with one’s self (SCS Self-Kindness), labeling internal experiences with words (FFMQ Describe) [56], awareness of, attention to and acceptance of one's painful experiences in a balanced and non-judgmental way (SCS Mindfulness), ability to refocus to positive, happy and pleasant thoughts instead of thinking about threatening and stressful events (CERQ Positive refocusing), ability to give a positive meaning to the adverse events in terms of personal growth (CERQ Positive reappraisal), ability to refocus on what to do and how to handle the experience one has had (CERQ Planning), the ability to remind ourselves that suffering is part of the human nature and that I am in-group despite of my negative characteristics (SCS Common Humanity) [57], and the ability to relativize the adverse event compared to other events, by using a broader focus (CERQ Perspective taking) [58]. There is an effort in the ST movement to integrate mindfulness to enhance the Healthy Adult mode [59–61], or self-compassion [62], and emotion regulation [19, 63]. We can say that there are strong associations between these concepts and schema modes based on our results.

This combination of adaptive schema modes, mindfulness, adaptive self-compassion, and adaptive cognitive emotion regulation strategies are in a reverse relationship with the first canonical variate pair's general personality pathology side. Based on this, we can reason that those therapeutic interventions that facilitate the development of these schema modes and adaptive emotion regulation strategies together may lead to changes in the general personality pathology, the other side of the canonical variate pair. Schema therapy has promising results in the treatment of a wide range of personality disorders [64].

The Non-adaptive side of the first canonical variate pair represents the general personality pathology, or 'p factor', with externalizing and internalizing pathology, which is an indicator of a broad predisposition to psychopathology. This finding is in accordance with the hierarchical models of psychopathology. For example, Krueger and Markon, in their meta-analyses of published studies of multivariate comorbidity models applied to DSM-defined dichotomous mental disorders in large, population-representative samples, found that meta-analytic estimate of the correlation between internalizing and externalizing factors is approximately .50 [65]. The non-adaptive side of our first canonical variate pair may be understood as an underlying transdiagnostic liability construct for internalizing, externalizing, and mixed personality pathology.

According to schema mode theory, if needs are not met, the patient is flipping between dissociative states of maladaptive modes. Schema modes that fit to internalizing pathology are the punitive or demanding internal dialogues (Punitive and Demanding Parent), painful negative emotions induced by frustrated needs, like fear, abandonment, shame, guilt, sadness (Vulnerable Child), submissive (Compliant Surrender) and depersonalizing (Detached Protector) avoidant coping modes. Externalizing schema modes are frustrated needs induced Impulsive/Undisciplined/Angry/Enraged Child modes and overcompensator Self-Aggrandizer and Bully-Attack coping modes. This conceptual division of schema modes was partly empirically proven in Jacobs et al. [35] study.

We grouped the non-adaptive emotion-regulation strategies that contributed to the first canonical variate pair into primarily internalizing and more general strategies. The primarily internalizing strategies were the harshly self-critical (SCS Self-Judgment), self-blaming (CERQ Self-blame) and non-accepting (DERS Nonaccept) reactions to one's distress, as well as the pervasive sense of isolation (SCS Isolation), furthermore the catastrophizing (CERQ Catastrophizing) and ruminative (CERQ Rumination, Jermann et al., 2008) thinking style. The more general emotion regulation strategies that may underlie both the internalizing and the externalizing pathology were the following: the belief that there is little that can be done to regulate emotions effectively once an individual is upset (DERS Strategy); difficulties engaging in goal-directed behavior, that is difficulties concentrating and accomplishing tasks when experiencing negative emotions (DERS Goals); difficulties remaining in control of one's behavior when experiencing negative emotions (DERS Impulse); the extent to which individuals know (and are clear about) the emotions they are experiencing and having a clear understanding of the nature of these responses (DERS Clarity); lack of emotional awareness, and inattention to, and lack of awareness of, emotional responses (DERS Awareness) [9]; being "over-identified" with thoughts and feelings so that we are caught up and swept away by negative reactivity (SCS Over-Identification) [57]. Several meta-analyses demonstrated that difficulties in emotion regulation and a lack of adaptive regulatory strategies, as well as a lack of self-compassion, are trans-diagnostic features of psychopathology [13, 20, 66]. This pattern of multivariate associations between schema modes and emotion regulation strategies may represent a latent general personality pathology profile, which may explain the high level of comorbidity between personality disorder and other mental disorder categories. Schema therapy specifically, but other evidence-based treatments of personality disorders, like Dialectical-Behaviour Therapy, Mentalization Based Therapy, Transference Focused Therapy, to mention the main methods, address some or all elements of this general personality pathology profile therapeutically.

The second canonical variate pair, which we dubbed "Externalizing," is a latent variable that may represent an underlying risk for multiple disorders within the externalizing spectrum. This pattern of multivariate associations between schema modes and emotion regulation strategies may lead to further understanding of the externalizing personality pathology. The 2. schema mode variate contains associations between the Impulsive/Enraged/Angry and Undisciplined Child modes, and the overcompensator, Self-Aggrandizer, and Bully Attack modes. These child modes are externalizing emotional reactions to unmet needs. The Self-Aggrandizer coping mode includes states of dominance, arrogance, and superiority, and the Bully and Attack coping mode includes the use of threats and aggression to intimate others or defend oneself against perceived threats.

The emotion regulation strategies related to the 2. emotion regulation variate may be grouped under three topics: disruptive consequences of negative emotions, blaming others, and a maladaptive form of self-kindness. Difficulties engaging in goal-directed behavior (DERS Goals) and difficulties in controlling impulses (DERS Impulse) when experiencing negative emotions and being "over-identified" with thoughts and feelings, so that the person is caught up and swept away by negative reactivity (SCS Over-Identification) were positively related to the externalizing schema modes. These externalizing personality profile elements in association with the Impulsive/Enraged/Angry and Undisciplined Child modes stress the importance of disruptive negative emotions, which fit the reactive externalizing subtype, characterized by a spontaneous lack of control that occurs with little if any thought, and leads to impulsive, affective, or hostile aggression. Our externalizing profile does not fit the proactively aggressive externalizing persons' profile, characterized by proactive, premeditated, predatory, or instrumental aggression [67].

Blaming others (CERQ) was most strongly associated with the 2. emotion regulation variate of the Externalizing canonical variate pair. Also, it is positively related to the externalizing schema modes. Furthermore, its association is very specific to the Externalizing variate pair. This result fits previous findings that blaming others is an attributional style of the externalization dimension [68]. Blaming others' positive relation to the Angry/Rageful Child mode may represent a form of other -condemning anger [69]. Similarly to our findings, Blaming others (CERQ) was positively correlated with the experience and expression of anger and inversely correlated with one form of adaptive anger control among college students [70]. Blaming others also positively relates to Self-Aggrandizer and Bully-Attack modes. These results fit previous findings that blaming others equally strongly correlated with relationship aggression among men and women [71], and it is most strongly and consistently associated with career criminality among confined delinquents [72].

Finally, Self-blame (CERQ), Self-Judgment (SCS) were inversely, and Self-Kindness (SCS) was positively related to the externalizing schema modes. This constellation of low self-blame and low self-judgment with high self-kindness triad among individuals struggling with self-aggrandizing and impaired self-control when experiencing negative emotion may have a consequence that they are less apt to monitor the social consequences of their behavior, and may be less prone to experience self-reflective emotions, like shame and guilt as an adaptive commitment device [73], which plays a role in preventing transgressive behavior. One of the elements of personality functioning in the alternative DSM-5 model for personality disorders is self-direction, which is the ability to self-reflect productively and use constructive and pro-social internal standards of behavior [74]. The appearance of this above-mentioned triad in the Externalizing variate pair in this sample of patients with personality disorders may represent a dysfunctional version of self-direction. In the schema mode model, therapy aims to facilitate the development of the Healthy Adult mode, which not just recognizes, validates, and asserts unmet core emotional needs of the vulnerable child, but also sets limits for the angry/rageful child and the impulsive/undisciplined child, and moderates the maladaptive coping modes, in accord with the principles of reciprocity and self-discipline [75]. In the Externalizing variate pair, the Healthy Adult mode is not playing a role. Without limit-setting capacity, the triad of the seemingly adaptive self-kindness and non-judgment, together with a low level of self-blaming may represent a less adaptive constellation of the self-monitoring system.

The emphasis on self-kindness, non-judgment, and self-acceptance, in addressing problems underlying this latent variable may be counterproductive. For example, mindfulness or self-compassion-based interventions targeting self-kindness and reducing self-judgments may lessen the adaptive function of shame and other self-evaluative mental processes that monitor the long-term social consequences of the individual's actions. Three interventions address this problem in schema therapy: limited reparenting, empathic confrontation, and behavior pattern breaking. In case of limited reparenting, the therapist tries to compensate for the patient's early unmet developmental needs within appropriate limits and boundaries, and one core developmental need is self-control and limit-setting. Empathic confrontation is a two-step technique. First, the therapist addresses early maladaptive schemas and dysfunctional mode behavior, with empathy for the frustrated need, which may have triggered them; second, the therapist confronts these maladaptive reactions as needing to change for the patient to have a healthy life. Schema therapy also uses specific techniques for confronting and setting limits on patients' inadequate coping behaviors and teaching more adaptive coping skills [76]. In sum, the Externalizing variate pair covers many aspects of the Aggression part of the Externalizing pathology identified by Krueger and South [77]. Interestingly, the Detached Protector schema mode, which fits the substance use part of the externalizing pathology did not relate substantially to any canonical variate pair.

Our third specific hypothesis, which was based on van Wijk-Herbrink et al. [78], was partially supported by our data. The adaptive CERQ scales negatively and the maladaptive scales positively related to the 1. emotion regulation variate. Two scales related to the 2. emotion regulation variate (negative loading for self-blame and positive loadings for blaming others). The Acceptance subscale was not associated with any of the variates. Based on van Wijk-Herbrink et al. we assumed that CERQ scales would form three distinct group: maladaptive (catastrophizing, rumination, and blaming others), adaptive (positive reappraisal, putting into perspective, positive refocusing, acceptance, and refocus on planning), and external attribution (negative loading for self-blame and positive loadings for positive refocusing and blaming others) emotion regulation. The differences between van Wijk-Herbrink's results and ours are that the acceptance subscale of CERQ did not have significant relations with any variates and that positive refocusing did not relate to the 2. emotion regulation variate. In addition, in our results, other-blame is only in the 2. emotion regulation variate. Finally, self-blame is in the 1. emotion regulation variate on the non-adaptive side. Based on our and Wijk-Herbrink’s results, low self-blame and high other blame may be an important part of externalizing pathology.

## Limitations

Limits of the current study needs to be acknowledged. These stem mainly from the cross-sectional nature of the research. Schema modes are not personality traits but intense emotional states that abruptly change during a relatively short period of time. The design of our study might assume that schema modes are trait-like phenomena characterized by specific emotion regulation strategies. A longitudinal study, e.g. Experience Sampling Method could have been a more appropriate research design to examine the schema mode that the subject is in at a given moment, and to reveal the emotion regulation strategies associated with this momentary state. A second limitation is related to the self-report questionnaires assessing schema modes and emotion regulation strategies, which might have distorted the data. Moreover, our sample is non-meditating, so the FFMQ and SCS items may have different meanings than in a meditator sample [56]. Another limit of our study is that 50 patients did not complete their SCID II interviews.

## Conclusion

Using a multivariate approach (CCA), we identified two independent patterns of multivariate associations between maladaptive schema modes and emotion regulation strategies. The Adaptive/Non-Adaptive general personality pathology profile includes adaptive and non-adaptive schema modes and emotion regulation strategies, confirming our hypothesis that adaptive schema modes are positively associated with other adaptive schema modes and adaptive emotion regulation strategies, while negatively associated with maladaptive schema modes and non-adaptive emotion regulation strategies. This profile represents a general personality pathology, or 'p factor', which is an indicator of a broad predisposition to psychopathology. The second, Externalizing personality pathology profile, which is independent from the previous one, can be interpreted as a pattern of multivariate associations between the Impulsive, Enraged, Angry, Undisciplined, Self-Aggrandizer and Bully Attack schema modes and emotion regulation strategies grouped under three topics: disruptive consequences of negative emotions, blaming others, and a maladaptive form of self-kindness. These two personality pathology profiles may lead to a deeper understanding of the associations between schema modes and emotion regulation strategies and underlying vulnerability dimensions of personality disorders. They may help psychotherapists in their conceptualization in order to design the most appropriate interventions.

## Supplementary Information

**Additional file 1.**

**Additional file 2.**

## Data Availability

The dataset analyzed during the current study is available as a Supporting File. The data is partly overlapping with the dataset we have used in another research [79], the patients who participate in both datasets are marked with yellow under the column “Code”.
